# Early detection of cardiotoxicity in pediatric and adolescent patients with cancer treated with anthracyclines in Northeastern Brazil

**DOI:** 10.1016/j.htct.2026.106255

**Published:** 2026-02-08

**Authors:** Jéssica Laureano Martins, Fabiana Gomes Aragão Magalhães Feitosa, Maria Verônica Câmara dos Santos, Thaysa Maria Gama Albuquerque Leão de Menezes, Andrea Dantas Sena, Edinalva Pereira Leite Rodrigues, Marcela Beatriz Alves Lopes, Júlia Laís dos Santos, Maria do Carmo Menezes Bezerra Duarte

**Affiliations:** a Instituto de Medicina Integral Prof. Fernando Figueira (IMIP), Recife, Pernambuco, Brazil; b Hospital Universitário Oswaldo Cruz (HUOC), Recife, Pernambuco, Brazil; c Sociedade Brasileira de Oncologia Pediátrica (SOBOPE), São Paulo, São Paulo, Brazil; d Faculdade Pernambucana de Saúde (FPS), Recife, Pernambuco, Brazil

**Keywords:** Cardiotoxicity, Echocardiography, Anthracyclines, Cancer, Children

## Abstract

**Objective:**

To evaluate the early detection of cardiotoxicity using echocardiography in children and adolescents with cancer treated with anthracyclines.

**Methods:**

This cross-sectional study was conducted in a tertiary pediatric oncology center in Northeastern Brazil between January 2018 and December 2022. Eligible participants were under 19-year-old patients with cancer treated with anthracyclines presenting left ventricular ejection fraction ≥55% (assessed using the biplane Simpson’s method) and abnormal left ventricular global longitudinal strain.

**Results:**

A total of 45 patients meeting the inclusion criteria were included. Among them, 19 patients (42.2%) showed reduced ejection fraction or left ventricular global longitudinal strain (or both) compared with baseline values, and 57.9% were asymptomatic. The most prevalent cancer was leukemia (55.5%), followed by lymphoma (20.0%). A total of 75.6% of participants were undergoing cancer treatment at the time of diagnosis of cardiotoxicity. An isolated left ventricular ejection fraction reduction occurred in 26.3% of patients, isolated left ventricular global longitudinal strain reduction in 47.4% of patients, and both alterations were experienced by 26.3% of patients.

**Conclusions:**

**A**nthracycline-induced cardiotoxicity is a relevant adverse effect in cancer treatment, especially in patients with leukemia and lymphoma. Echocardiography, especially the assessment of left ventricular global longitudinal strain, plays a critical role in the early and subclinical detection of cardiotoxicity in pediatric patients.

## Introduction

Cancer during childhood accounts for about 3 % of all malignant neoplasms. According to estimates by the Brazilian National Cancer Institute, 7930 new pediatric cancer cases are expected annually during the 2023–2025 triennium [1]. Leukemia (28.0 %), central nervous system tumors (26.0 %), and lymphomas (8.0 %) are among the most prevalent types [2]. Advances in treatment have led to favorable outcomes, with survival rates reaching about 80 % in developed countries [2,3]. The increased survival has enhanced awareness of treatment-related adverse effects, particularly those affecting long-term health [4,5].

Cardiovascular complications are prevalent during cancer treatment. Cardiotoxicity is diagnosed during or after treatment by cardiovascular alterations, clinical findings, biomarkers, or imaging tests occurring after excluding other etiologies [6]. The clinical manifestations may range from asymptomatic to heart failure, arrhythmias, arterial hypertension, pericarditis, thromboembolism, or myocardial ischemia [4,6,7].

Anthracyclines are among the most widely used chemotherapeutic agents for treating pediatric cancer [2,6,[8], [9], [10]]. However, their use is closely associated with cardiovascular effects, particularly myocardial dysfunction [6,[8], [9], [10], [11]]. Classic risk factors for cardiotoxicity include age (especially below five years), cumulative dose (CD) of anthracycline ≥250 mg/m², underlying heart disease, Down syndrome, electrolyte disturbances, mediastinal irradiation, cardiotoxic drugs, and female sex [2,6,8,10]. The pathophysiology of anthracycline-related cardiotoxicity is multifactorial, involving mitochondrial damage, enzymatic alterations, oxidative stress resulting from the production of free radicals, and the inhibition of topoisomerase II, which reduces myocardial contractility and leads to myocyte death [2,3,6,10,12,13].

Echocardiography is used for non-invasive monitoring of myocardial function during and after cancer treatment. The biplane Simpson’s method for measuring left ventricular ejection fraction (LVEF) is widely recommended for assessing systolic function [6,7,11,14]. In addition, left ventricular global longitudinal strain (LVGLS) has emerged as a sensitive indicator for the early detection of subclinical myocardial dysfunction [2,11,12,[14], [15], [16], [17], [18], [19]]. This study aimed to evaluate the early detection of cardiotoxicity using echocardiography in children and adolescents with cancer treated with anthracyclines.

## Methods

This cross-sectional study was conducted using secondary data retrieved from the medical records of patients treated at the pediatric cardio-oncology outpatient clinic of Hospital Universitário Oswaldo Cruz (HUOC) located in Recife (Pernambuco, Brazil) between January 2018 and December 2022. The study included under 19-year-old cancer patients treated with anthracyclines who had preserved LVEF (assessed using the biplane Simpson's method) and at least one previous echocardiogram showing abnormal LVGLS [14]. Exclusion criteria were applied and patients who did not undergo a cardiological evaluation during treatment; presented with previous left ventricular dysfunction (LVD) such as an LVEF <55 % [14,20]; did not start chemotherapy at the institution; had a single echocardiogram; or had no LVGLS during follow-up were excluded. Only patients with at least one abnormal baseline LVGLS were included, since the study specifically aimed to monitor the evolution and early detection of subclinical myocardial dysfunction among those with initial subclinical impairment.

The variables studied included age at the onset of cancer treatment, sex, type of cancer, risk factors for cardiotoxicity, treatment initiation and completion, anthracycline CD, and patient status at the time of data collection. Left ventricular systolic function was analyzed using LVEF and LVGLS. According to current guidelines in pediatric cardio-oncology, the values considered abnormal were LVEF <55.0 % and the absolute value of LVGLS <−18 % [14,[21], [22], [23], [24]]. A LVGLS between −16 % and −17 % was considered borderline*,* and LVD was described by levels higher than −16 % [14].

Cardiotoxicity was diagnosed echocardiographically as an asymptomatic reduction in LVEF of ≥10.0 % compared with baseline, or a reduction below normal limits (<55.0 %), or LVGLS with a reduction of ≥15.0 % compared with baseline [[6], [7], [8]]. Echocardiograms were performed by the same operator using the same equipment (CX 50 Philips® SLGVE software aCMQ 10.5.1 and Affinity 50 Philips® software aCMQ 11.0). All patients were hemodynamically stable during the echocardiogram.

Participants were divided into two groups. The overall cohort consisted of 45 eligible patients with available longitudinal data on LVEF (at baseline and at the time of dysfunction) and reduced LVGLS values. A subgroup of 19 patients was classified based on evidence of cardiotoxicity [[6], [7], [8]].

A descriptive analysis of the data was performed. Absolute and relative frequencies described categorical variables, while numerical variables were expressed as mean and standard deviation or median and interquartile range. The study was approved by the research ethics committee of the Instituto de Medicina Integral Prof. Fernando Figueira under the opinions 66126722.8.0000.5201 and 66126722.8.3001.5192 of HUOC. The Strobe checklist for observational studies was applied [25].

## Results

**Fig. 1:**
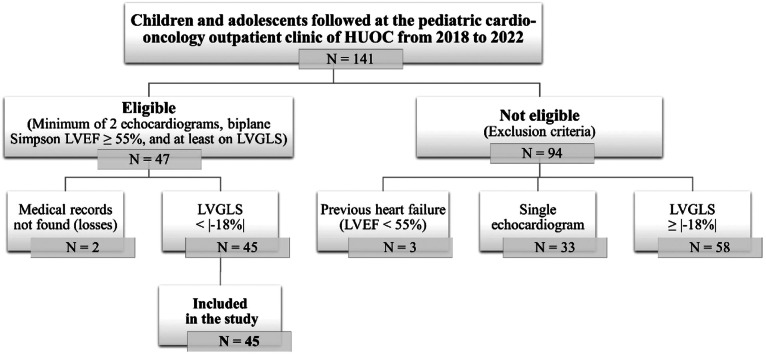
Flowchart of the recruitment of study participants. HUOC: Hospital Universitário Oswaldo Cruz; LVEF: left ventricular ejection fraction; LVGLS: left ventricular global longitudinal strain.

A total of 141 children and adolescents were followed up at the pediatric cardio-oncology outpatient clinic of HUOC between 2018 and 2022 (Figure 1). Of these, 45 out of 141 patients (31.9 %) were included in this study, and 19 out of 45 patients (42.2 %) presented cardiotoxicity.

Table 1, Table 2 present the clinical variables of the full sample (*n* = 45) and the subgroup (*n* = 19), respectively. The mean age at cancer diagnosis was 10.3 ± 4.5 years, ranging from 1 to 17 years. Acute lymphoblastic leukemia was the most prevalent type of cancer among eligible patients. Of the full sample, 75.5 % of patients had hematologic cancers, comprising 55.5 % with leukemias and 20.0 % with lymphomas; the prevalence of hematologic cancers in the subgroup was 68.4 %.

**Table 1:** Clinical and echocardiographic description of the 45 patients with evolutionary analysis of LVEF and primary reduction in LVGLS.

Patient (No.)	Sex	Age (years)	Type of cancer	Baseline LVEF / TT* ( %)	LVGLS in TT* ( %)	Δ DX CTX (months)	Status
1	F	8	Osteosarcoma	80 / 28	−13	101	OT
2	M	10	ALL	71.3 / 62	−17.4	7.5	In treatment
3	F	6	Wilms' tumor	59 / 56	−17	4	In treatment
4	M	14	Ewing's sarcoma	66 / 51	−16	10	In treatment
5	M	14	ALL	56.7 / 58	−15	14.5	In treatment
6	F	10	ALL	58 / 51	−14.8	5	Death (sepsis)
7	F	13	AML	59 / 60	−15	6	In treatment
8	M	9	ALL	65 / 53	−16.8	8.5	OT
9	F	17	Sarcoma	68 / 72	−17	3	In treatment
10	F	11	NHL	62 / 65	−16.6	2.5	In treatment
11	M	16	NHL	62.2 / 55	−17	5	Death (sepsis)
12	F	7	ALL	60 / 49.3	−12.5	3	Death (disease progression)
13	M	17	LH	60 / 50	−15	3	In treatment
14	F	4	ALL	65.6 / 58	−17.3	49	OT
15	F	14	ALL	65 / 60	−16.3	5	In treatment
16	M	3	Retinoblastoma	71 / 40	−11	63	Death (disease progression)
17	F	1	ALL	67 / 52	−14	11	Death (disease progression)
18	M	9	ALL	63 / 55	−16.5	23	OT
19	M	12	Ewing's sarcoma	61 / 50	−15.4	5.5	Death (HF)
20	M	14	Ewing's sarcoma	62 / 57	−17.2	16	Death (disease progression)
21	M	13	Osteosarcoma	65 / 60	−15	3.5	Death (disease progression)
22	M	15	ALL	60 / 55	−16	1	In treatment
23	M	15	ALL	65 / 60	−17.4	3	In treatment
24	M	8	HL	60 / 58	−14	2	In treatment
25	M	16	ALL	60 / 58	−17	21.5	In treatment
26	M	6	HL	61.4 / 57	−15.6	0.3	In treatment
27	M	1	Retinoblastoma	65.3 / 62	−17	21.5	OT
28	F	17	HL	59 / 61	−16.5	7	In treatment
29	M	11	ALL	66 / 60	−16.7	11	In treatment
30	F	15	ALL	64.2 / 63	−17	1	In treatment
31	M	16	ALL	65 / 70	−17	10	In treatment
32	M	14	ALL	55 / 55	−16	19	In treatment
33	M	14	ALL	60.3 / 55	−16	32	In treatment
34	F	14	HL	61 / 61	−16	11.5	OT
35	M	4	ALL	65 / 61	−17	49.5	OT
36	M	12	Osteosarcoma	58 / 68	−17.2	4.5	In treatment
37	F	8	ALL	57.5 / 64.8	−17.6	0.25	In treatment
38	F	7	ALL	68 / 62	−17	18	OT
39	F	18	ALL	60 / 55	−14.8	0.5	In treatment
40	F	7	ALL	65 / 61	−17.8	51	OT
41	F	10	HL	58 / 63.2	−17.8	8	OT
42	M	16	Sarcoma	60 / 55	−12.3	4	In treatment
43	M	6	ALL	62.4 / 62.4	−16.2	3	In treatment
44	F	10	ALL	60.5 / 55	−16.2	1	In treatment
45	F	17	HL	66 / 67	−17	5	In treatment

F: Female; M: Male; ALL: acute lymphoblastic leukemia; AML: acute myeloid leukemia; HL: Hodgkin’s lymphoma; NHL: non-Hodgkin lymphoma; LVEF: left ventricular ejection fraction; LVGLS: left ventricular global longitudinal strain*;* Δ: time differential; DX: diagnosis; CTX: cardiotoxicity; OT: off therapy; HF: heart failure. *TT: time of acquisition of LVEF and LVGLS during or after treatment. Status: clinical situation of the patient at the time of data acquisition and death (cause of death).

**Table 2:** Clinical and echocardiographic description of the progression of the reduction ≥10 percentage points in LVEF and/or ≥15 % in LVGLS in relation to baseline values.

Patient No.	Sex	Age (years)	Type of cancer	Anthracycline CD (mg/m²)	Baseline ECHO (LVEF/LVGLS) ( %)	ECHO CTX (LVEF/LVGLS) ( %)	LVEF pp reduction / % LVGLS reduction	Δ ECHO basal x CTX (months)	Status
1	F	8	Osteosarcoma	450	80 / −18	28 / −13	52 pp / 27.8 %	101	OT
2	M	10	ALL	180	71.3 / −21.8	62 / −17.4	20.2 % LVGLS	7.5	In treatment
3	F	6	Wilms' tumor	100	59 / −21	56 / −17	19 % LVGLS	4	In treatment
4	M	14	Ewing's sarcoma	375	66 / −18	51 / −14	15 pp / 22.2 %	10	In treatment
5	M	14	ALL	300	56.7 / −19.4	58 / −15	22.7 % LVGLS	14.5	In treatment
6	F	10	ALL	345	66 / no	51 / −14.8	15 pp LVEF	5	Death (sepsis)
7	F	13	AML	560	59 / −19.7	60 / −15	23.8 % LVGLS	6	In treatment
8	M	9	ALL	270	65 / −22	53 / −16.8	12 pp / 23.6 %	8.5	OT
9	F	17	Sarcoma	150	68 / −20	72 / −17	15 % LVGLS	3	In treatment
10	F	11	NHL	60	62 / −20.5	65 / 16.6	19 % LVGLS	2.5	In treatment
11	M	16	NHL	340	62.2 / −23.7	55 / −17	28.3 % LVGLS	5	Death (sepsis)
12	F	7	ALL	180	60 / −23	49.3 / −12.5	10.7 pp / 45.6 %	3	Death (disease progression)
13	M	17	HL	120	60 / −18	50 / −15	10 pp / 16.7 %	3	In treatment
14	F	4	ALL	300	65.6 / −21	58 / −17.3	17.6 % LVGLS	49	OT
15	F	14	ALL	150	65 / −20.8	60 / −16.3	21.6 % LVGLS	5	In treatment
16	M	3	Retinoblastoma	242	71 / no	40 / −11	31 pp LVEF	63	Death (disease progression)
17	F	1	ALL	340	67 / no	52 / −14	15 pp LVEF	11	Death (disease progression)
18	M	9	ALL	300	73 / −18.5	63 / −16.5	10 pp LVEF	23	OT
19	M	12	Ewing's sarcoma	300	61 / −18	50 / −15.4	11 pp LVEF	5.5	Death (HF)

F: Female; M: Male; ALL: acute lymphoblastic leukemia; AML: acute myeloid leukemia; NHL: non-Hodgkin lymphoma; CD: cumulative dose; ECHO: echocardiogram; LVEF: left ventricular ejection fraction; LVGS: left ventricular global longitudinal strain; CTX: cardiotoxicity*;* pp: percentage points; Δ ECHO: time differential between baseline and CTX diagnosis; Status: clinical status of the patient at the time of data acquisition and death (cause of death); OT: off therapy; HF: heart failure.

Regarding the timing of cardiotoxicity diagnosis, 75.6 % of the full sample were undergoing anthracycline treatment (Table 1). Similarly, 14 patients (73.7 %) from the subgroup developed cardiotoxicity during chemotherapy, while five (26.3 %) were not undergoing therapy (Table 2). Secondary causes for LVD were excluded in all patients undergoing cancer treatment.

In the subgroup, 52.6 % of the patients were female, 15.8 % were under 5 years of age, 5.3 % had Down syndrome, and the anthracycline CD was >250 mg/m² in 57.9 % of the cases (61.5 % had leukemia and lymphoma). No patient underwent mediastinal radiotherapy, and 57.9 % were asymptomatic for cardiovascular conditions.

A total of 10 out of 45 patients showed a reduction of ≥10 % in LVEF compared with baseline, and 35 did not have a significant change in LVEF. Of these, 31 patients (68.9 %) had borderline LVGLS, and 14 (31.1 %) met the criteria for LVD (Table 1). In the subgroup, the analysis of cardiotoxicity was progressive and confirmed by the echocardiographic criteria (Table 2). Baseline LVEF values ranged from 56.7 to 80.0 % in this group, and the post-treatment levels from 28.0 to 72.0 %. Meanwhile, the pre-treatment LVGLS values ranged from −18.0 to −23.7 %, and the post-treatment values ranged from −11.0 to −17.4 %. The mean time between baseline echocardiographic evaluation and the onset of cardiotoxicity was 17.3 months. Table 3 summarizes the echocardiographic findings of the subgroup. An isolated reduction in LVEF was observed in five patients (26.3 %), an isolated LVGLS reduction in nine patients (47.4 %), and a concurrent reduction in both parameters in five patients (26.3 %). During the follow-up period, 17.8 % of patients died. Causes of death included disease progression (11.1 %), sepsis (4.4 %), and heart failure (2.2 %).

**Table 3:** General echocardiographic evolution of the 19 patients with cardiotoxicity.

Echocardiographic variables	n ( %)
LVEF	5 (26.3)
LVGLS	9 (47.4)
Both	5 (26.3)
**Baseline values -** median (range)	
LVEF	65 (56.7 to 80)
LVGLS	−20.2 (−18 to −23.7)
**Altered values -** median (range)	
LVEF	55 (28 to 72)
LVGLS	−15.4 (−11 to −17)

LVEF: left ventricular ejection fraction; LVGLS: left ventricular global longitudinal strain.

## Discussion

Anthracyclines are essential chemotherapeutic agents with relevant efficacy in treating cancer during childhood. However, their use offers limitations due to substantial cardiovascular complications. The risk of cardiovascular mortality in survivors is about eightfold higher than in the general population [8].

This study describes the clinical characteristics and echocardiographic findings of 45 children and adolescents with cancer treated with anthracyclines presenting normal LVEF and reduced LVGLS at diagnosis. Nineteen patients (42.2 %) developed a reduction of ≥10.0 % in LVEF or ≥15.0 % in LVGLS compared with baseline and consequent cardiotoxicity diagnosis.

Among patients who developed cardiotoxicity, classical risk factors described in pediatric cardio-oncology, including female sex, age under five years, and Down syndrome, were proportionally more prevalent, consistent with previous studies [6,8,10].

Among the subgroup of 19 patients with cardiotoxicity, 57.9 % received an anthracycline CD >250 mg/m². The average time between baseline and abnormal echocardiograms was 17.3 months, and the mortality rate was 31.6 %. Studies in Colombia, France, and Germany also associated high doses of anthracyclines with increased risk of cardiotoxicity [3,24,26]. However, ventricular dysfunction has also been observed with lower doses [2]. In the present study, 42.2 % of patients presented cardiotoxicity, regardless of the time between exams and despite the CD being lower than 250 mg/m², which reinforces the notion that anthracyclines do not have a safe dose.

The high prevalence of hematological cancers in the present study (34 out of 45 patients - 75.6 %), with a predominance of acute lymphoblastic leukemia, is consistent with Brazilian epidemiology [1]. In the subgroup, 68.4 % had leukemia and lymphoma, and 61.5 % (8 out of 13 patients) were exposed to anthracycline CD >250 mg/m². These findings underscore the relevance of enhanced cardiologic monitoring in pediatric patients with hematologic cancers due to the increased risk of anthracycline-induced cardiotoxicity and the consequent decreased quality of life of survivors [24,26].

Most cardio-oncology studies with children involve cancer survivors [10]. However, early detection of subclinical cardiotoxicity and intervention is crucial to prevent the progression of myocardial injury [2,10,16]. Linares et al. prospectively evaluated 112 patients with acute leukemia aged between 1 and 18 years [26]. The prevalence of early cardiotoxicity (first week to one year after the end of cancer treatment), identified by echocardiographic values of LVEF and LVGLS, was 17.9 %.

Early diagnosis plays a critical role in the management of cardiotoxicity. In this study, most patients were undergoing chemotherapy (75.6 %). Among the 19 patients with relevant heart failure, 73.7 % were diagnosed with cardiotoxicity during treatment, but 57.9 % of this subgroup was asymptomatic, and 47.4 % presented preserved LVEF with isolated reductions in LVGLS (Table 1), underscoring the role of early diagnosis in subclinical injury.

Wolf et al. retrospectively evaluated 79 pediatric patients with cancer who were treated with anthracyclines for ten years. Cases of acute leukemia and Hodgkin's lymphoma were the most prevalent among the sample, and 28 % had preserved LVEF but abnormal LVGLS (absolute value <−20.0 %).

The retrospective study by Rique et al. evaluated 38 children with acute leukemia treated with anthracyclines and detected alterations in LVGLS in 28.9 % of the cases, using the cutoff value of −20.0 % proposed by Levy et al. [22,24]. These studies emphasized the clinical relevance of early detection of cardiotoxicity, as the inclusion of the assessment in LVGLS in clinical practice was recommended [24]. However, the present study utilized the updated cutoff established by Mertens et al. in 2023 for pediatric cardio-oncology, defining the LVGLS criterion for LVD as an absolute value ≤−16.0 % (borderline LVGLS) [14].

Similarly, Gunsaulus et al. reported that an abnormal LVGLS during anthracycline treatment predicts future cardiotoxicity in children, confirming the prognostic importance of early strain alterations [27].

In cases where the baseline LVGLS was unavailable, Almeida et al. suggested that absolute values lower than −17.0 %, excluding other cardiac diseases, may indicate subclinical cardiotoxicity [11]. In this study, patients with an isolated reduction of LVEF without baseline LVGLS had post-treatment values of −11.0 %, −14.0 %, and −14.8 % (Table 2). Therefore, baseline assessment also plays a critical role in the early diagnosis of cardiotoxicity.

Current Brazilian, European, and North American cardio-oncology and cardiovascular imaging guidelines recommend echocardiography as the first-line modality for cardiovascular monitoring during cancer treatment [2,6,7,14,28]. In this study, most patients with abnormal LVGLS remained asymptomatic. Even in cases with preserved LVEF, reduced LVGLS values demonstrated the ability to detect subclinical myocardial dysfunction. The inclusion of patients with abnormal baseline LVGLS focused the analysis on early progression of subclinical myocardial injury, in accordance with the sensitivity of LVGLS for detecting anthracycline-related dysfunction even in the presence of normal LVEF [14,27,28].

This study had some limitations. First, it was conducted at a single center, which may affect the external validity of the findings. However, HUOC is a university referral center for pediatric oncology in Northeast Brazil, exclusively serving the Brazilian public healthcare system. Second, the retrospective design restricted access to some clinical data (e.g., socioeconomic data, comorbidities, anthracycline CD in all patients, and use of other chemotherapy drugs and dexrazoxane). Although dexrazoxane is part of the institutional protocol, no documentation was found in the medical records consulted. Additionally, the diastolic function was not evaluated. Last, the inclusion criterion of abnormal LVGLS likely contributed to an increased prevalence of cardiotoxicity, given the focus on the sensitivity of LVGLS even with preserved LVEF.

Although the retrospective design and small sample size limited generalizability, the internal consistency of the findings was reinforced by the fact that all echocardiograms were performed by a single operator using standardized equipment.

The findings of this study underscore the relevance of baseline echocardiographic assessment of LVEF and LVGLS for the early diagnosis of subclinical cardiotoxicity in pediatric patients with cancer treated with anthracyclines, allowing for timely cardiologic intervention and potentially improving their quality of life [[28], [29], [30]].

## Conclusion

Anthracycline-induced cardiotoxicity is a relevant adverse effect during cancer treatment, especially in patients with leukemia and lymphoma. Early detection of cardiotoxicity in children and adolescents treated with anthracyclines is crucial to ensure the safety and efficacy of cancer treatment. Incorporating LVGLS into routine clinical practice in pediatric oncology should be encouraged, given its high sensitivity in detecting subclinical myocardial injury and its well-established prognostic value. Furthermore, baseline assessment of LVEF and LVGLS is essential for accurate diagnosis, effective monitoring, and comprehensive evaluation of cardiac function before, during, and after cancer treatment**.**

## Author's contributions

Martins JL: Conceptualization, Formal analysis, Investigation, Writing – review and editing. Feitosa FGAM and dos Santos MVC: Conceptualization, Investigation, Writing – review and editing. Menezes TMGAL and Sena AD: Conceptualization, Formal analysis, Writing – original draft. Rodrigues EPL: Data acquisition, Investigation, Writing – review and editing. Lopes MBA and Santos JL: Data acquisition, Investigation. Duarte MCMB: Conceptualization, Formal analysis, Investigation, Writing – review and editing. All authors approved the final version and declared no conflict of interest.

## Funding

None

## Conflicts of interest

The authors declare no conflict of interest to report.

## Data Availability

The data that support the findings of this study are available from the corresponding author upon reasonable request.
